# Macular Pucker: A Devastating Complication in Ocular Toxoplasmosis

**DOI:** 10.7759/cureus.34617

**Published:** 2023-02-04

**Authors:** Syarifah Nur Humaira Syed Mohd Khomsah, Julieana Muhammed, Wan-Hazabbah Wan Hitam

**Affiliations:** 1 Department of Ophthalmology and Visual Science, School of Medical Sciences, Universiti Sains Malaysia, Kota Bharu, MYS

**Keywords:** headlight in the fog, macular pucker, toxoplasmosis, vitrectomy, partial thickness macula hole, ocular toxoplasmosis

## Abstract

Ocular toxoplasmosis has multiple devastating complications with possible recurrence. A potentially blinding complication of ocular toxoplasmosis is macular pucker. Here, we report a case of macular pucker in ocular toxoplasmosis treated with azithromycin and prednisolone. A 35-year-old woman complained of central scotoma for six days, which was associated with fever, headache, joint pain, and myalgia. Her visual acuity was counting finger OD and 6/18 OS. Her right eye optic nerve function test was impaired. Fundoscopy showed bilateral optic disc swelling that progressed to retinal fibrosis over papillomacular bundle and macular pucker over the right eye. CT scan of the brain and orbit was normal. Toxoplasma titer was positive. She was diagnosed to have a right eye macular pucker secondary to ocular toxoplasmosis. Oral azithromycin and oral prednisolone (on a tapering dose) were administered for six weeks. Fundoscopy showed resolved optic disc swelling. However, her vision in the right eye remained poor. Ocular toxoplasmosis may progress to macular pucker which can lead to poor vision and legal blindness. Reduced vision-related quality of life notably in the younger population as a complication of ocular toxoplasmosis is difficult to prevent. However, therapy with azithromycin and prednisolone may reduce the negative consequences of inflammation and shrink lesions, especially when the lesions are located at the macula or near the optic disc. Vitrectomy is an alternative treatment for complications such as macular pucker in selected cases.

## Introduction

Toxoplasmosis is an infection of the opportunistic parasite *Toxoplasma gondii* (*T. gondii*) which is prevalent worldwide and has a primary feline host. The infection in chronic or latent form is estimated to vary from 10% to 50% of the population in Malaysia [1]. Ocular toxoplasmosis is an ocular inflammation and a potentially blinding disease with recurrences as the posterior segment is typically affected and is the most significant cause of vision disability in children and adults. An anti-retinal autoimmune pathway that triggers retinal damage has been postulated [2]. The currently available treatment for this disease involves halting inflammation and reducing complications. The objective of this paper is to highlight the clinical finding of macular pucker as a complication of ocular toxoplasmosis.

## Case presentation

A 35-year-old woman with no underlying comorbidity came to the emergency department with a complaint of high-grade fever for nine days. It was also associated with headache, joint pain, myalgia, and productive cough without a history of bleeding tendencies and rashes. The patient had a right eye central scotoma for six days which was noticeable while closing one eye and progressively worsening. Otherwise, she denied eye pain, eye redness, and retro-orbital pain. The patient did not have a history of contact with a pulmonary tuberculosis patient nor a history of recent traveling; however, she had a pet unvaccinated cat at home with a history of being scratched one month ago. She reported no history of constitutional symptoms, no family history of malignancy, and no drug or food allergies. Upon the next review in the clinic, she developed floaters bilaterally.

**Figure 1:**
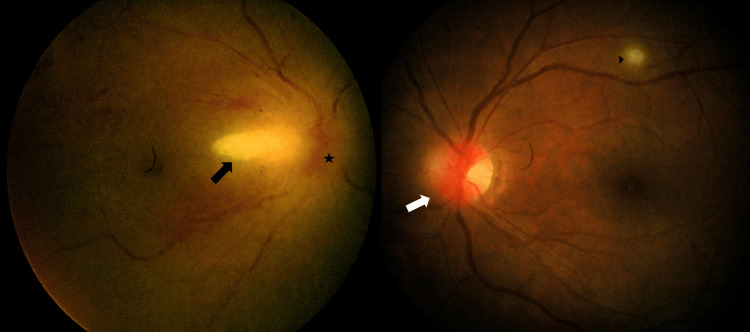
Right hyperemic optic disc with ill-defined margin (star) and area of retinitis temporal to the disc (black arrow), left hyperemic and swollen optic disc nasally (white arrow), and area of retinitis (arrowhead).

On examination, the patient was alert and conscious. Her right eye vision was counting fingers 1 feet, and the left eye was 6/18, pinhole 6/18. Her right eye reverse afferent pupillary defect was found positive. The Ishihara test result was 0/15, light brightness was 2/10, and red saturation was 0/15. Her left eye optic nerve function test was normal. Her extraocular muscle movement was normal bilaterally, and she was found with a central scotoma over her right eye during confrontation. Her anterior segment was normal in both eyes. The right fundus showed a 360-degree swollen, elevated, and hyperaemic disc with an ill-defined margin and vitritis (Figure 1).

**Figure 2:**
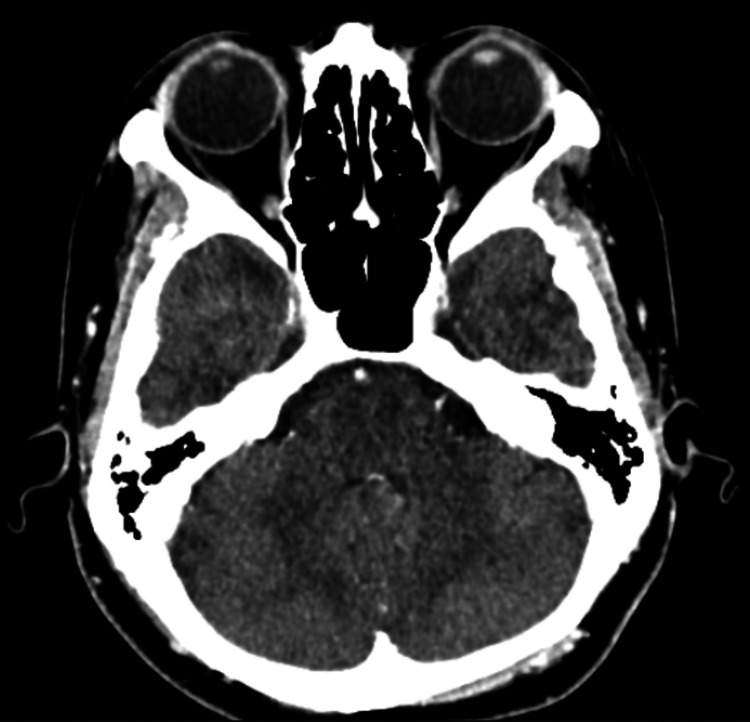
Contrasted CT of the brain revealed no focal or enhancing brain parenchymal lesion and no evidence of a space-occupying lesion.

An urgent contrasted CT of the brain and orbit revealed normal findings (Figure 2).

Moreover, the erythrocyte sedimentation rate (ESR) and C-reactive protein (CRP) were raised. Her full blood count and renal profile were within normal range. Mantoux test was negative, and she had normal chest X-ray findings. Toxoplasma serology IgG was positive with a titer of 246.4 IU/mL while IgM was negative. Serology for hepatitis B, hepatitis C, HIV, and syphilis were negative. Cytomegalovirus IgG was positive with a titer of 1,377 U/mL, and the connective tissue screening was found negative.

The patient was diagnosed with bilateral ocular toxoplasmosis with differentials of cat scratch disease or bilateral ocular tuberculosis. She was started on oral azithromycin 500 mg once daily (OD) for six weeks and oral prednisolone 40 mg OD tapering dose for six weeks.

**Figure 3:**
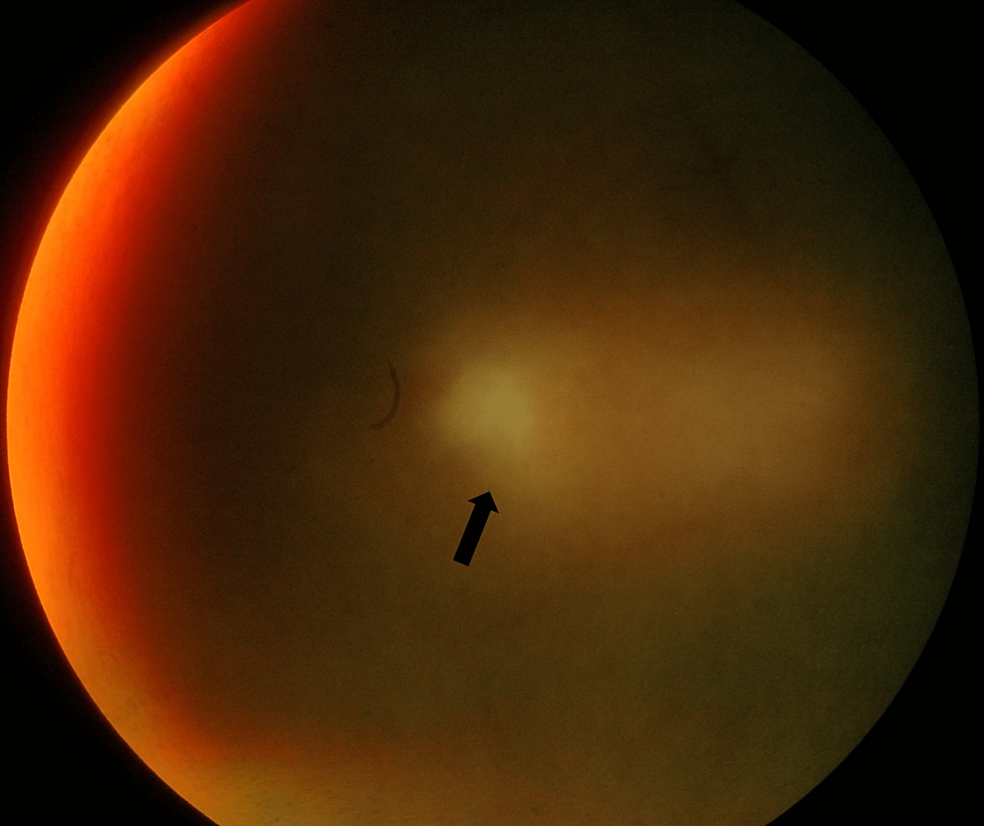
Headlight in the fog; severe vitritis with an ill-defined area of retinitis (black arrow).

The patient was followed up every week initially for one month, followed by every two weeks for two months. In the first week, after starting the treatment, the patient had worsening clinical findings with increasing floaters over the right eye and severe vitritis with macula edema which was not apparent during the initial presentation (Figure 3).

**Figure 4:**
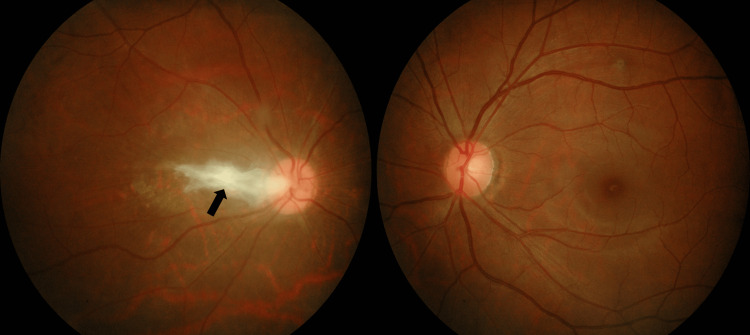
Right eye fibrosis at the papillomacular bundle with traction on the macula eight weeks after the treatment (black arrow).

At the end of the treatment, her vision was hand movement over the right eye and 6/18 with pinhole 6/7.5 over the left eye. The fundus showed right eye fibrosis involving the papillomacular bundle with macular puckering (Figure 4).

**Figure 5:**
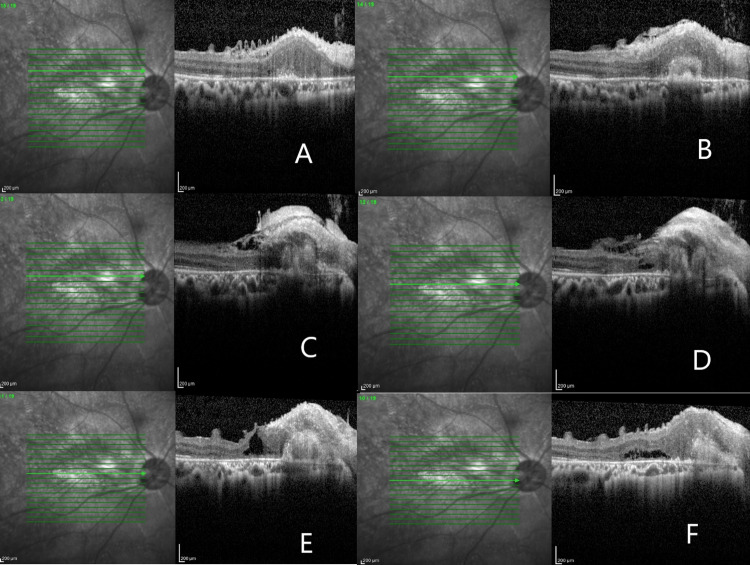
A-C: Inner retinal fibrosis at the papillomacular bundle. D: Intraretinal fluid; E: Partial thickness macula hole with traction on the macula/macular puckering. F: Subretinal fluid.

The optical coherence tomography (OCT) of the right eye showed areas of inner retinal disorganization and fibrosis with macular puckering (Figure 5).

## Discussion

Toxoplasmosis is the most common cause of posterior uveitis in the world, and, in some countries, it accounts for more than 80% of cases [3]. The current finding for seroprevalence of toxoplasmosis in immunocompetent individuals in Malaysia ranged from 13.9% to 30.2%. In comparison with other ethnic groups, Malays showed the highest prevalence, and a higher incidence was found in males and unemployed individuals, while a lower rate was noted in people with higher incomes [1]. In pregnant women, 23-49% of toxoplasma prevalence was found in Malaysia. Ocular toxoplasmosis occurs in 2-18% of patients who are seropositive [4].

The routes *T. gondii* uses to infect humans are from ingestion of oocyst-contaminated food or water, intake of undercooked meat containing tissue cysts, cured meat products, sashimi (Japanese raw fish dish) served in sushi restaurants, and transplacental passage of tachyzoites during primary maternal infection. However, rarely, infections are also transmitted by organ transplantation. Contact with infected felines and rodents, frequent gardening, and intestinal parasitic infections are other risk factors [5]. In this case, the patient had close contact with a cat, and a positive history of cat scratches put her at risk for toxoplasmosis.

*T. gondii* may enter the human retina by leucocytes carrying the parasite or by transmigrating tachyzoites crossing the vascular endothelium and direct infection of endothelial cells. By crossing the human neuroretina, the parasite may reach various cell types. The most susceptible host cells are retinal Müller glial cells. When the retinal pigment epithelial cells are infected with *T. gondii*, it affects growth factor production and causes the neighboring uninfected epithelial cells to proliferate. The distinctive hyperpigmented toxoplasmic retinal lesion may result from an increase in the sensitivity of cells to parasite infection. Additionally, infected epithelial cells cause a strong immunologic reaction and have an impact on the activity of leukocytes infiltrating the retina [6].

Active ocular toxoplasmosis typically presents as a retinochoroiditis, with focal necrotizing granulomatous retinitis, reactive granulomatous choroiditis, vitritis, and iridocyclitis, along with vision-threatening complications such as retinal detachment, choroidal neovascularization, and glaucoma [7]. In this patient, the fundus showed a 360-degree swollen and hyperaemic disc with an ill-defined margin. The area of choroiditis was seen at the inferotemporal arcade with vascular sheathing and vitritis highly suggestive of the possibility of ocular toxoplasmosis. The acute presentation of posterior uveitis and “headlight in the fog” fit the clinical feature of ocular toxoplasmosis; however, the late complication of inner retinal fibrosis involving the papillomacular bundle and partial thickness macula hole with macular puckering was a relatively rare sight-threatening complication in ocular toxoplasmosis.

Seropositivity against *T. gondii* infection and the presence of antibodies are relatively high worldwide and are only useful for checking prior parasite exposure. Diagnosis of ocular toxoplasmosis cannot be confirmed only by this seropositive finding. Therefore, a high index of suspicion of clinical presentations is the greatest clue for diagnosis. This patient had a right eye posterior uveitis and bilateral optic disc swelling coupled with elevated *T. gondii *antibody titers which were used to verify the disease.

A potentially toxic treatment with pyrimethamine is indicated in the event of active sight-threatening disease, and azithromycin is a non-toxic antibiotic administered in patients who are unable to tolerate the effect of pyrimethamine. It penetrates phagocytic cells and reaches high intracellular and tissue concentrations. In vivo and in vitro efficacy against *T gondii *has been reported, with an effect on the cystic form if administered for longer than four weeks [8]. In this patient, oral azithromycin 500 mg OD for six weeks was used to avoid the potentially toxic treatment of triple therapy. The vitritis and acute inflammatory reaction resolved at week four of treatment. The patient was able to tolerate the antibiotic well with the concurrent treatment of prednisolone. However, the vision was still poor post-treatment due to the fibrosis of the inner retina involving the papillomacular area. 

Several cases have reported that vitrectomy may be useful to address complications such as full-thickness macula holes, secondary epimacular membranes, and retinal detachment [9-12]. However, in this patient, as there was disorganization and fibrosis of the inner retinal layers at the papillomacular bundle found on OCT, vitrectomy may not result in adequate benefit for the patient. After thorough preoperative examinations and detailed discussions of benefits, risks, and alternatives with the patient, a vitrectomy was not done as she was not keen on surgery.

## Conclusions

In immunocompetent patients, a devastating complication may arise even after adequate treatment, and recurrence is unavoidable due to the *T. gondii* cyst being resistant to all forms of anti-toxoplasma medication. Treatment for ocular toxoplasmosis can only alleviate the inflammation and halt retinal damage in sight-threatening cases. Although it is one of the most common causes of blindness secondary to posterior uveitis in children and young adults, there is still no definitive cure for it. Treatment with azithromycin has minimal side effects compared to other anti-toxoplasma medications such as triple therapy. Vitrectomy has a role in treating ocular toxoplasmosis complications, such as macula hole, epiretinal membrane, macular pucker, and retinal detachment.
